# Prospective study of grapefruit intake and risk of breast cancer in postmenopausal women: the Multiethnic Cohort Study

**DOI:** 10.1038/sj.bjc.6603880

**Published:** 2007-07-10

**Authors:** K R Monroe, S P Murphy, L N Kolonel, M C Pike

**Affiliations:** 1 Department of Preventive Medicine, Keck School of Medicine, University of Southern California, Los Angeles, CA 90089-9175, USA; 2 Cancer Research Center of Hawaii, University of Hawaii, Honolulu, HI 96813, USA

**Keywords:** breast cancer, grapefruit intake, CYP3A4 metabolism

## Abstract

*In vitro* and *in vivo* studies have shown that cytochrome *P*450 3A4 (CYP3A4) is involved in the metabolism of oestrogens. There is evidence that grapefruit, an inhibitor of CYP3A4, increases plasma oestrogen concentrations. Since it is well established that oestrogen is associated with breast cancer risk, it is plausible that regular intake of grapefruit would increase a woman's risk of breast cancer. We investigated the association of grapefruit intake with breast cancer risk in the Hawaii–Los Angeles Multiethnic Cohort Study, a prospective cohort that includes over 50 000 postmenopausal women from five racial/ethnic groups. A total of 1657 incident breast cancer cases were available for analysis. Grapefruit intake was significantly associated with an increased risk of breast cancer (relative risk=1.30, 95% confidence interval 1.06–1.58) for subjects in the highest category of intake, that is, one-quarter grapefruit or more per day, compared to non-consumers (*P*_trend_=0.015). An increased risk of similar magnitude was seen in users of oestrogen therapy, users of oestrogen+progestin therapy, and among never users of hormone therapy. Grapefruit intake may increase the risk of breast cancer among postmenopausal women.

The inhibitory effect of grapefruit juice on the intestinal cytochrome *P*450 3A4 (CYP3A4) system was discovered accidentally in 1989 during a study designed to test the effect of ethanol on a calcium-channel blocker (Bailey *et al*, 1989, 1991). Grapefruit juice was given to subjects to mask the taste of ethanol. Subsequent investigations found that through the inhibition of the CYP3A4 enzyme system, primarily in the liver and small intestine (Guengerich, 1999; Veronese *et al*, 2003), grapefruit juice interacts with more than 60% of orally administered drugs leading to elevation of their serum concentrations (Maskalyk, 2002; Bailey and Dresser, 2004). Consumption of a single glass (six ounces) can produce the maximal acute pharmacokinetic effect (Edgar *et al*, 1992; Lundahl *et al*, 1995, 1997; Dahan and Altman, 2004) with enhanced oral drug bioavailability occurring up to 24 h after juice consumption (Bailey and Dresser, 2004).

Since 1989, the list of drug interactions with grapefruit juice has expanded to include oral 17*β*-oestradiol and progesterone (Guengerich, 1999; Maskalyk, 2002; Medical Letter, 2005). The US Food and Drug Administration (FDA) mandated labelling for hormone products for postmenopausal women now contains warnings that grapefruit juice may increase plasma concentrations of oestrogen (US Food and Drug Administration, 2006). Schubert *et al* (1994) found that grapefruit juice increased the area under the curve of oestradiol approximately 20% in ovariectomised women. Recently, we reported that endogenous oestrogen levels were about 30% higher in postmenopausal women in whom periods had stopped naturally and who were consuming the equivalent of ¼ grapefruit or more per day (Monroe *et al*, 2007).

It is well established that oestrogen is associated with breast cancer risk (Key *et al*, 2002). Therefore, if grapefruit intake affects oestrogen metabolism leading to higher circulating levels, then it is biologically plausible that regular intake of grapefruit would increase a woman's risk of breast cancer. To our knowledge, no study has yet examined this hypothesis. The present study investigated the association of grapefruit intake and breast cancer risk in the Hawaii–Los Angeles Multiethnic Cohort (MEC) Study.

## MATERIALS AND METHODS

### Study population

The MEC was assembled in 1993–1996 when 96 810 adult men and 118 441 adult women, aged 45–75 at baseline, completed a 26-page, self-administered mailed questionnaire that contained a quantitative food frequency questionnaire (FFQ) and information on demographic factors, personal behaviours, prior medical conditions, use of medications, family history of common cancers, and, for women, reproductive history and use of oral contraceptives and hormone therapy (HT). The study was approved by the Institutional Review Boards of the University of Hawaii and the University of Southern California. Further details regarding the study have been given elsewhere (Kolonel *et al*, 2000).

Only women from the five racial/ethnic groups – African Americans, Japanese Americans, Latinas, Native Hawaiians, and Caucasians – were included in the study reported here. Also we only included women who reported that their menstrual periods had stopped naturally or due to oophorectomy. Women who had undergone hysterectomy but reported one or more ovaries still intact were excluded. We also excluded women with missing or invalid data for weight, height, oestrogen and/or progestin use, age at menarche, parity, age at first live birth, age at natural menopause or age at bilateral oophorectomy, physical activity, smoking status, or education level. We also excluded subjects if they had been diagnosed with cancer other than non-melanoma skin cancer before cohort entry. Finally, we excluded subjects if their intake of calories or its components (fat, protein, and carbohydrate) from the baseline FFQ fell outside a defined valid range (Nothlings *et al*, 2005). As a result, data for 46 080 postmenopausal women were available for analysis.

### Surveillance

Incident cases of breast cancer in California were identified by linkage to the Los Angeles County SEER registry and to the State of California Cancer Registry (CCR). Incident cancers in Hawaii were identified using the statewide Hawaii SEER registry and the CCR was used to identify cases who had moved to California. A software program was used to conduct a probabilistic data linkage using social security number, name, and date of birth. We had a social security number on 98.7% of the cohort. Further, we determined that after an average period of 7 years in the cohort, the out-migration rate was 3.7% (4.9% for Hawaii participants and 2.5% for California participants). Although the out-migration rate is somewhat higher for Hawaii, California is the primary destination for these migrants, so that most remain under surveillance for cancer incidence. Mortality was determined by annual linkage to state death files in California and Hawaii, and periodically to the National Death Index. Follow-up time began on the date the respondent completed the baseline questionnaire and, for the analysis reported here, continued through 31 December 2002. Subjects were censored at the date of last follow-up, date of death, or date of diagnosis of cancer, whichever occurred earlier.

### Assessment of grapefruit intake from baseline questionnaire

The methods for dietary assessment have been described in detail elsewhere (Kolonel *et al*, 2000). The FFQ asked respondents to report how often they ate ‘grapefruit or pomelo’ during the past year and the usual portion size. Usual intake was reported by marking one of the following eight frequencies: never or hardly ever; once a month; 2–3 times a month; once a week; 2–3 times a week; 4–6 times a week; once a day; 2 or more times a day. Portion sizes for whole grapefruit included ¼ cup or less, ½ grapefruit or ½ cup, and 1 cup or more. The average weight of one grapefruit was estimated to be 240 g. Total grapefruit intake was calculated by multiplying the frequency and portion size and expressed as grams per day. We were unable to quantify intake of grapefruit juice as it was combined with orange juice in the FFQ. The performance of the FFQ in a calibration sub-study has been previously reported (Stram *et al*, 2000).

### Menopausal hormone therapy use from baseline questionnaire

Subjects were asked about use of menopausal oestrogen therapy (ET) and progestin therapy (PT) separately in the baseline questionnaire. Oestrogen+progestin therapy (EPT) was calculated based on the overlap between reported periods of ET and PT use (Lee *et al*, 2006). For the present analysis, we used a categorical variable defined as follows: (1) no HT use; (2) past HT use; (3) current ET use; and (4) current EPT use.

### Statistical analysis

Hazard ratios for breast cancer incidence, referred to in this report as relative risks (RRs), were estimated using log-linear regression proportional hazard models stratified on age at recruitment (single year), year of recruitment (single year), race/ethnicity (African American, Japanese, US-born Latina, Latina born in Mexico or other Central or South American country, Native Hawaiian, and Caucasian), and study centre (Hawaii/California). We examined grapefruit intake by categories of both absolute intake (none, <30 g, <60 g, and ⩾60 g) and intake per 1000 kcal (none, <15, <30, and ⩾30 g per 1000 kcal). The highest category of absolute intake (⩾60 g) was an average of slightly greater than ¼ grapefruit per day or ½ every other day. The highest category of intake density was roughly equivalent. Categories were determined by usual serving size (as defined above) from the FFQ and the intake distribution for all subjects. Non-dietary factors considered as potential confounders included age at menarche (⩽12, 13–14, and 15+), age at first live birth (⩽20, 21–30, and 31+), parity (nulliparous, 1 child, 2–3 children, and 4+ children), age at and type of menopause (natural menopause at age <45, 45–49, 50–54, and 55+ or oophorectomy at age <45, 45–49, and 50+), postmenopausal oestrogen and/or progestin use (as defined above), weight (continuous) and height (continuous) at recruitment, physical activity (continuous variable for hours per day engaged in vigorous work and/or strenuous exercise), family (mother or sister) history of breast cancer (yes/no), education level attained (⩽10th grade, high school, some college or vocational school, and college graduate), and smoking status at baseline (never, former, and current). Dietary factors considered as potential confounders included intakes of alcohol, total energy, saturated fat, total dietary fibre, and soluble and insoluble fibre fractions. The dietary fibre value used in the present study is that published in the USDA tables and is the sum of lignin and non-starch polysaccharides in plant foods (Prosky *et al*, 1985). The values for insoluble and soluble fractions were obtained by the Englyst procedure, which aims to measure plant cell wall non-starch polysaccharides as the sum of the chemically identified constituent sugars; the procedure does not measure lignin (Englyst *et al*, 1994). Although these two methods of fibre analysis are highly correlated, they are nevertheless distinct from one another and may not be combined. The variables included in the final multivariate model were established risk factors and factors we had found to influence serum oestrogen levels in our previous study (Monroe *et al*, 2007), that is, dietary fibre and soluble fibre intakes. The underlying time variable in the analysis was time from the date of cohort enrollment to the date of breast cancer diagnosis, death, or censoring.

Calculations were performed using the SAS (SAS Institute, Cary, NC, USA) and STATA (Stata Corporation, College Station, TX, USA) statistical software packages. Testing for significance was performed using likelihood ratio methods. All *P*-values quoted are two-sided.

## RESULTS

A total of 1657 of the women in the defined cohort were diagnosed with incident breast cancer before 31 December 2002 and are included in the analysis. Some baseline characteristics of the breast cancer cases and the total study population are shown in Table 1. Japanese Americans comprise the largest group with 32% of the population followed by Caucasians with 26%. Overall, whole grapefruit was consumed by 50% of the study population with 7% reporting intake of ¼ grapefruit or more per day. Grapefruit was consumed by 55% of Japanese Americans, 51% of African Americans, 49% of Caucasians, 45% of Latinas, and 35% of Hawaiian study subjects.

Table 2 shows the risk of breast cancer across category of grapefruit intake quantified by absolute intake (g) and by intake density (g per 1000 kcal). The first relative risk (RR_1_) estimate was adjusted for (stratified on) age at recruitment, year of recruitment, race/ethnicity, and study centre. Intake of ¼ grapefruit or more per day is associated with a 21% increase in breast cancer risk (RR_1_=1.21, 95% confidence interval (CI)=1.00–1.46, *P*_trend_=0.044); the highest category of grapefruit density (⩾30 g per 1000 kcal) is also associated with increased breast cancer risk (RR_1_=1.18, 95% CI=0.98–1.42, *P*_trend_=0.040). Second relative risk (RR_2_) estimate was adjusted (stratified) as above, and further adjusted for weight, height, physical activity, education, age at menarche, age at first birth, parity, age at and type of menopause, menopausal oestrogen/progestin use, cigarette smoking, family history of breast cancer, alcohol, saturated fat, total dietary fibre, and soluble fibre. With these adjustments, intake of ¼ grapefruit or more per day is significantly associated with a 30% increase in breast cancer risk (RR_2_=1.30, 95% CI=1.06–1.58, *P*_trend_=0.015); the highest category of grapefruit density is likewise associated with a 25% increase in breast cancer risk (RR_2_=1.25, 95% CI=1.03–1.52, *P*_trend_=0.013). The increase in RR from RR_1_ to RR_2_ was due to adjustment for total dietary fibre and soluble fibre; adjustment for insoluble and soluble fibre produced almost identical results (data not shown).

The associations between grapefruit intake and breast cancer risk by category of HT use are shown in Table 3. The reference category for all RR estimates is never HT/non-consumers of grapefruit. Because the results for grapefruit intake density were similar to results for absolute intake, we present only the latter. Among never HT users, there is a statistically significant positive association between the highest category of grapefruit intake (⩾60 g per day) and breast cancer risk (RR=1.44, 95% CI=1.08–1.93, *P*_trend_=0.038). There is no trend among past HT users. For current ET users, there is an increase in risk of 36% from the lowest (zero) category of intake to the highest (*P*_trend_=0.22); for current EPT users, breast cancer risk increases 27% from the lowest to the highest category of grapefruit intake (*P*_trend_<0.13). There was a dose–response relationship between grapefruit intake and breast cancer risk in never HT users and in current ET and EPT users. Although the trend tests are not significant for current ET and EPT users, the pattern is the same in the three groups, and there is no statistical evidence that the magnitudes of the trends differ.

We examined whether the association of grapefruit intake and breast cancer risk was modified by body mass index (BMI: weight in kilograms/(height in metres)^2^). Subjects were divided into two categories: lean/normal weight (BMI<25.0) and overweight/obese (BMI⩾25.0). The association between grapefruit intake and breast cancer risk was not modified by BMI, as shown in Table 4. The reference category for all RR estimates is BMI<25.0/non-consumers of whole grapefruit. Among lean/normal weight subjects, there is a statistically significant increased risk of breast cancer associated with grapefruit intake. Risk increases 32% from the lowest (zero) category of intake to the highest (*P*_trend_=0.011). Among overweight/obese subjects, breast cancer risk increases 26% from the lowest to the highest category (RR=1.46, 95% CI=1.10–1.96), although the test for trend is not statistically significant.

## DISCUSSION

To our knowledge, this is the first report of a commonly consumed food that may increase the risk of breast cancer among postmenopausal women. Whole grapefruit intake was significantly associated with breast cancer in the present study – generally, a 30% increase among subjects who consume the equivalent of ¼ grapefruit or more per day.

To reduce measurement error in dietary assessments, investigators often energy-adjust intakes by calculating densities, that is, g per 1000 kcal of total energy intake. Densities represent the contribution of a particular nutrient or food group in relation to overall dietary intake as measured by the FFQ (Willett, 1998). However, individual food intakes, particularly with respect to fruits and vegetables, are also commonly examined in terms of absolute or crude intake (van Gils *et al*, 2005). The biologic implication of the two analytic approaches needs careful consideration when interpreting diet–disease associations (Willett, 1998). We considered this issue in our data and found that both approaches produced similar results. We present the detailed results in terms of absolute intake to make them more easily interpretable.

The measure of grapefruit intake in the present study, based on intake of the whole fruit, is an underestimation of total intake owing to a lack of information on grapefruit juice. If one makes the assumption that grapefruit juice intake is highly correlated with whole fruit intake, then the magnitude of risk observed in the present study is biased upwards; it represents the risk of breast cancer owing to total intake not just whole fruit intake. However, if there is little correlation between whole grapefruit and grapefruit juice intakes, and if one accepts that grapefruit juice intake increases breast cancer risk in the same manner as the whole fruit, then the magnitude of risk observed in the present study may be an underestimation of the true risk. This requires further study.

Beginning in the 1980s, it was hypothesised that dietary fibre may have a beneficial effect on breast cancer incidence by lowering endogenous oestrogen levels through the partial interruption by dietary fibre of the enterohepatic recirculation of oestrogens (Goldin *et al*, 1982, 1986; Adlercreutz, 1990). While recent epidemiological evidence supports a role for dietary fibre intake in relation to lower endogenous hormone levels (Rock *et al*, 2004; Prentice *et al*, 2006), the question remains unsettled. In our study of endogenous oestrogen levels in postmenopausal women, oestradiol decreased monotonically from 10.6 to 6.2 pg ml^−1^ from the lowest to the highest quintile of dietary fibre intake (median intake of 9.9–21.7 g per 1000 kcal), whereas soluble fibre, after adjustment for dietary fibre, was associated with an increase in oestradiol from 6.8 to 11.5 pg ml^−1^ from the lowest to the highest quintile of intake (median intake of 2.9–7.1 g per 1000 kcal) (Monroe *et al*, 2007). Taken together, these results suggested that the component of total dietary fibre not accounted for by soluble and insoluble fibre was strongly negatively associated with serum oestradiol while soluble fibre increased oestradiol levels. This is complex as most plant foods contain a mixture of soluble and insoluble fibre, as well as other constituents. We hypothesised that the result for total dietary fibre represented high lignin-containing foods as lignin is the primary constituent of dietary fibre not accounted for by soluble and insoluble fibre. Further, there is evidence that lignin adsorbs oestrogen, more so than other fibre components (Shultz and Howie, 1986; Arts *et al*, 1991).

In the present study, the addition of total dietary fibre and soluble fibre to the statistical model modified the estimate of breast cancer risk associated with grapefruit intake (RR_1_ to RR_2_ in Table 2). On the basis of our observation that intakes of total dietary fibre and soluble fibre are significantly associated with serum oestrogen levels in naturally postmenopausal women, and the *in vitro* evidence that certain fibre components, particularly lignin, have the ability to adsorb oestrogen, the adjustment will provide a more accurate estimate of breast cancer risk associated with grapefruit intake.

Consistent with other studies on postmenopausal oestrogen use, we found an increase in breast cancer risk, particularly among current EPT users. One aim of our study was to examine the risk of breast cancer associated with grapefruit intake by subgroup of menopausal HT use. As endogenous oestrogens are metabolised in the same manner as exogenous estrogens (US Food and Drug Administration, 2006), we had hypothesised that grapefruit intake would increase a woman's risk of breast cancer, regardless of HT use. Support for the hypothesis among women taking HT was provided by the warning in FDA mandated labelling of menopausal HT products that grapefruit juice may increase plasma concentrations of oestrogens.

The association between grapefruit intake and breast cancer risk was clearly seen in never HT users, as well as current ET and EPT users. Interestingly, the RR of breast cancer associated with consumption of ¼ grapefruit or more per day compared to non-consumers was 44% higher among women who had never used HT, 36% higher in current ET users, and 27% higher among current EPT users. The risk of breast cancer associated with consumption of grapefruit was 32% higher among lean/normal weight women and 26% higher among overweight/obese women. Taken together, these results for HT and BMI suggest that the risk of breast cancer associated with grapefruit intake is stronger for subgroups of women with lower circulating oestrogen levels.

This finding of a reduced effect of grapefruit in women with a higher BMI is similar to the lower effect of ET on breast cancer risk in women with a higher BMI (Collaborative Group on Hormonal Factors in Breast Cancer, 1997; Schairer *et al*, 2000; Million Women Study Collaborators, 2003; Beral *et al*, 2005). The lower RR associated with ET in heavier women can be explained by the elevated endogenous oestrogen level in heavier women, and detailed analysis suggests that the ceiling of effective oestrogen as regards the breast is achieved by women with a BMI of approximately 32 kg m^−2^ (Wu *et al*, 2007). In the same way, increased oestrogen levels from grapefruit intake may have less and less effect as the ‘baseline’ oestrogen levels increase. Because the ‘baseline’ oestrogen level of women on ET or EPT is greater than the effective oestrogen ceiling (Wu *et al*, 2007), the increased risk from grapefruit intake in these women may be partly a long-term effect of increased progesterone levels in the premenopausal period, but this remains to be studied.

Previous studies have reported that the magnitude of interaction between a drug and grapefruit juice is markedly variable among individuals (Bailey *et al*, 1993, 1995; Lown *et al*, 1997; Bailey and Dresser, 2004). Subjects with the highest intestinal expression of CYP3A4 showed the greatest increase in plasma drug concentration of felodipine after grapefruit juice administration (Lown *et al*, 1997). Currently, there is no way to predict the potential interaction; such studies are urgently needed.

In our study of grapefruit intake and serum oestrogen levels, we observed an approximately 30% increase in oestrone and a 10% increase in oestradiol levels among women who consumed the equivalent of ¼ grapefruit or more per day. In the present study, we observed a 25–30% increase in breast cancer risk in the highest category of grapefruit intake. The meta-analysis of the relationship between levels of endogenous sex hormones and breast cancer risk in postmenopausal women by the Endogenous Hormones and Breast Cancer Collaborative Group (Key *et al*, 2002) found that a doubling of oestradiol was associated with an RR of breast cancer of 1.29 (95% CI=1.15–1.44). These results suggest that we should have observed a much smaller increase in breast cancer risk in the present study. Even if one uses the 30% increase in oestrone, then we should have found about a 12% increase in risk. The reason for the greater breast cancer risk than would be predicted solely on the basis of postmenopausal oestrogen levels may have a number of possible explanations. First, it may be due to chance since the CI in the present study includes such a value. Second, the estimated 30% increase in oestrone levels from grapefruit intake may be an underestimate because of misclassification of true dietary intake and because it does not include grapefruit juice. Third, grapefruit may influence oestrogen and progesterone levels in the premenopausal period and the larger effect we see is partly a carry-over effect. Other explanations are possible; more work is certainly needed.

It is biologically plausible that grapefruit intake may increase the risk of breast cancer among postmenopausal women. However, the results of the present study need to be confirmed in other studies with more comprehensive measures of grapefruit intake that include both the whole fruit and grapefruit juice.

## Figures and Tables

**Table 1:** Baseline characteristics of breast cancer cases and the total study population

	**Cases**	**Total population**
No. of subjects	1657	46 080
Age at cohort entry (in years), mean (s.d.)	62.1 (7.27)	61.5 (7.35)
Body mass index, mean (s.d.)	25.5 (5.20)	25.6 (5.28)
		
*Race/ethnicity*		
African American	253	8,398
Japanese American	579	14 906
Latina	248	8404
Native Hawaiian	144	2608
Caucasian	433	11 764
		
*Education*		
⩽10th grade	217	7063
High school	487	14 183
Some college or vocation school	482	13 585
College graduate	471	11 249
		
*Age at menarche*		
⩽12	834	22 138
13–14	642	18 151
15+	181	5791
		
*Age at first live birth*		
Never	271	5939
⩽20	343	12 046
21–30	888	24 923
31+	155	3172
		
*Parity*		
Nulliparous	271	5939
1 child	202	5012
2–3 children	750	20 537
4+ children	434	14 592
		
*Type of and age at (in years) menopause*		
Natural, < 45	167	5346
Natural, 45–49	392	11 177
Natural, 50–54	601	15 612
Natural, 55+	198	4148
Oophorectomy, < 45	152	5395
Oophorectomy, 45–49	92	2898
Oophorectomy, 50+	55	1504
		
*Postmenopausal HT use*		
Never	631	20 959
Former HT use	259	8436
Current ET use	207	5843
Current EPT use	560	10 842
		
*Smoking status*		
Never smoker	908	25 938
Former smoker	535	13 811
Current smoker	214	6331
		
*Family history of breast cancer*		
Yes	271	5005
No	1386	41 075
		
*Alcohol intake*		
None	977	28 556
<12 g per day (one drink)	466	12 876
<24 g per day	82	2108
⩾24 g per day	132	2540
		
*Grapefruit intake*		
None	794	23 203
<30 g per day	635	16 911
<60 g per day	95	2692
⩾60 g per day (¼ grapefruit)	133	3274

EPT=oestrogen+progestin therapy; ET=oestrogen therapy; HT=hormone therapy.

**Table 2:** Relative risks (with 95% confidence intervals) of breast cancer across categories^a^ of grapefruit intake

*Grapefruit (g per day)*					
Intake	None	>0 to <30 g	⩾30 to <60 g	⩾60 g	*P*_trend_
No. of cases	794	635	95	133	—
Adjusted RR_1_	1.00	1.08 (0.97–1.21)	1.04 (0.83–1.29)	1.21 (1.00–1.46)	0.044
Adjusted RR_2_	1.00	1.08 (0.97–1.20)	1.07 (0.86–1.34)	1.30 (1.06–1.58)	0.015
*Grapefruit (g per 1000 kcal per day)*					
Intake	None	>0 to <15 g	⩾15 to <30 g	⩾30 g	—
No. of cases	794	632	93	138	—
Adjusted RR_1_	1.00	1.08 (0.97–1.20)	1.12 (0.90–1.40)	1.18 (0.98–1.42)	0.040
Adjusted RR_2_	1.00	1.07 (0.96–1.19)	1.17 (0.94–1.47)	1.25 (1.03–1.52)	0.013

RR_1_=first relative risk; RR_2_=second relative risk.

RR_1_ stratified on age at recruitment, year of recruitment, race/ethnicity, and study centre.

RR_2_ stratified as above and adjusted for weight, height, physical activity, age at menarche, age at first birth, parity, age at and type of menopause, oestrogen and/or progestin use, smoking, education, family history of breast cancer, alcohol, total energy, saturated fat (g per 1000 kcal), dietary fibre (g per 1000 kcal), and soluble fibre (g per 1000 kcal).

a Categories for grapefruit intake are based on total grams for 1 grapefruit=240 g per day. The highest category therefore represents ¼ grapefruit every day or ½ every other day.

**Table 3:** Relative risks (with 95% confidence intervals) of breast cancer across categories^a^ of grapefruit intake by HT use

**Grapefruit (g per day)**	**0**	**>0 to <30 g**	**⩾30 to <60 g**	**⩾60g**	***P*_trend_**
*Never HT*					
No. of cases	298	244	30	59	—
Adjusted RR_1_	1.00	1.19 (1.00–1.41)	0.87 (0.59–1.27)	1.44 (1.08–1.93)	0.038
					
*Past HT*					
No. of cases	133	91	17	18	—
Adjusted RR_1_	1.22 (0.99–1.50)	1.13 (0.88–1.43)	1.44 (0.87–2.37)	1.17 (0.72–1.92)	0.97
*Current ET*					
No. of cases	125	109	19	22	—
Adjusted RR_1_	1.56 (1.21–2.01)	1.70 (1.29–2.23)	1.85 (1.06–3.25)	2.12 (1.29–3.49)	0.22
					
*Current EPT*					
No. of cases	238	191	29	34	—
Adjusted RR_1_	2.01 (1.69–2.40)	2.09 (1.73–2.51)	2.48 (1.72–3.58)	2.55 (1.80–3.63)	0.13

EPT=oestrogen+progestin therapy; ET=oestrogen therapy; HT=hormone therapy; RR_1_=first relative risk.

RR_1_ stratified on age at recruitment, year of recruitment, race/ethnicity, and study centre and adjusted for weight, height, physical activity, education, age at menarche, age at first birth, parity, age at and type of menopause, smoking, family history of breast cancer, alcohol, total energy, saturated fat (g per 1000 kcal), dietary fibre (g per 1000 kcal), and soluble fibre (g per 1000 kcal).

a Categories for grapefruit intake are based on total grams for one grapefruit=240 g per day. The highest category therefore represents ¼ grapefruit every day or ½ every other day.

**Table 4:** Relative risks (with 95% confidence intervals) of breast cancer across categories^a^ of grapefruit intake by BMI

**Grapefruit (g per day)**	**0**	**>0 to <30 g**	**⩾30 to <60 g**	**⩾60 g**	***P*_trend_**
*BMI*<25.0					
No. of cases	407	339	55	74	
Adjusted RR_1_	1.00	1.05 (0.90–1.22)	1.14 (0.85–1.52)	1.32 (1.02–1.72)	0.011
					
*BMI⩾25*					
No. of cases	387	296	40	59	
Adjusted RR_1_	1.16 (1.00–1.34)	1.30 (1.11–1.52)	1.16 (0.83–1.62)	1.46 (1.10–1.96)	0.113

BMI=body mass index; RR_1_=first relative risk.

RR_1_ stratified on age at recruitment, year of recruitment, race/ethnicity, and study centre and adjusted for oestrogen/progestin use, physical activity, education, age at menarche, age at first birth, parity, age at and type of menopause, smoking, family history of breast cancer, alcohol, total energy, saturated fat (g per 1000 kcal), dietary fibre (g per 1000 kcal), and soluble fibre (g per 1000 kcal).

a Categories for grapefruit intake are based on total grams for one grapefruit=240 g per day. The highest category therefore represents ¼ grapefruit every day or ½ every other day.
